# Validation of the Japanese version of the Esophageal Hypervigilance and Anxiety Scale for esophageal symptoms

**DOI:** 10.1007/s00535-024-02193-w

**Published:** 2024-12-09

**Authors:** Akinari Sawada, Yoshimasa Hoshikawa, Hiroko Hosaka, Masahiro Saito, Hirotaka Tsuru, Shunsuke Kato, Eikichi Ihara, Tomoyuki Koike, Toshio Uraoka, Kunio Kasugai, Katsuhiko Iwakiri, Daniel Sifrim, John Erik Pandolfino, Tiffany H. Taft, Yasuhiro Fujiwara, Japan EHAS Study Group, Fumio Tanaka, Masaki Ominami, Tadashi Ochiai, Kei Yamamoto, Yuki Hisaki, Shiko Kuribayashi, Hideaki Itami, Kazuma Yachi, Yukihiro Shuto, Yoshitaka Hata, Masafumi Wada, Shinya Izawa, Yasushi Funaki

**Affiliations:** 1 Department of Gastroenterology, Graduate School of Medicine, Osaka Metropolitan University, Osaka, Japan; 2 Department of Gastroenterology, Nippon Medical School Graduate School of Medicine, Tokyo, Japan; 3 Department of Gastroenterology and Hepatology, Gunma University Graduate School of Medicine, Gunma, Japan; 4 Division of Gastroenterology, Tohoku University Graduate School of Medicine, Miyagi, Japan; 5 Department of Medicine and Bioregulatory Science, Graduate School of Medical Sciences, Kyushu University, Fukuoka, Japan; 6 Department of Gastroenterology, Aichi Medical University Graduate School of Medicine, Aichi, Japan; 7 Wingate Institute of Neurogastroenterology, Blizard Institute, Barts and The London School of Medicine and Dentistry, Queen Mary University of London, London, UK; 8 Division of Gastroenterology and HepatologyNorthwestern Medicine, Feinberg School of Medicine, Northwestern University, Evanston, IL USA; 9 The Rome Foundation Research Institute, Chapel Hill, Raleigh, NC USA

**Keywords:** Esophageal Hypervigilance and Anxiety Scale, High-resolution manometry, Esophageal symptoms, Esophageal motility disorders, Achalasia

## Abstract

**Background:**

The Esophageal Hypervigilance and Anxiety Scale (EHAS) is an English questionnaire created in the USA to assess these factors in all patients with esophageal diseases. The aim of this study was to develop and validate the Japanese version of EHAS and investigate the relationship between EHAS scores and symptoms in untreated disorders of esophagogastric junction (EGJ) outflow.

**Methods:**

This prospective study recruited patients who underwent high-resolution manometry (HRM) at six tertiary centers in Japan. The EHAS was translated to Japanese using standard forward and backward translation methods. Patients completed the following questionnaires: the Japanese EHAS, Eckardt score, Gastroesophageal Reflux Disease Questionnaire, and Hospital Anxiety and Depression Scale for assessment of construct validity. Logistic regression analysis identified factors associated with esophageal symptom severity in untreated disorders of EGJ outflow.

**Results:**

Overall, we analyzed 432 patients. Their main symptoms were dysphagia and reflux. The most common HRM diagnosis was normal (35.9%), followed by achalasia (29.4%). The Japanese EHAS demonstrated excellent reliability, and construct validity, with two subscales similar to the original EHAS. Total EHAS score moderately correlated to Eckardt score (*r* = 0.545, *p* < 0.001). In 113 patients with untreated disorders of EGJ outflow, multivariable analysis demonstrated that younger age, type II achalasia, and higher EHAS score were independently associated with higher Eckardt score.

**Conclusions:**

The Japanese EHAS is a reliable and valid questionnaire. Its subscale scores can be used as in the original version with some caution. Future studies are warranted to assess the appropriateness of factor loading.

**Supplementary Information:**

The online version contains supplementary material available at 10.1007/s00535-024-02193-w.

## Introduction

Esophageal symptoms such as reflux, dysphagia, and chest pain are common gastrointestinal (GI) symptoms. The estimated prevalence of reflux symptoms reaches approximately 20% in Japan [1–3]. In addition, heartburn and dysphagia account for more than 1.2 million annual ambulatory visits in the USA [4]. In most cases, these symptoms originate from benign esophageal disorders including gastroesophageal reflux disease (GERD), esophageal disorders of gut–brain interaction (DGBI), esophageal motility disorders, and eosinophilic esophagitis (EoE). It is conceivable that they share a common mechanism regarding symptom perception despite each disorder having its own primary etiology.

In general, the generation of esophageal symptoms begins with the activation of peripheral nerve endings in the esophageal wall. Subsequently, through the vagal and/or spinal nerve pathways, the activation is conveyed to the central nervous system where the signal is perceived as an esophageal symptom. Nerve sensitivity plays a crucial role in modulating the intensity of the signals [5], which can be altered by both peripheral and central factors. Peripheral factors include mucosal integrity [6, 7], cytokines and prostaglandin E2, superficial mucosal afferent nerves [8, 9], and sensory receptors. Central factors encompass sleep disturbance [10–13] and psychological factors [14, 15].

Pertaining to psychological factors, the severity of heartburn and chest pain in patients with GERD positively correlates with symptom-specific anxiety, rather than acid burden (i.e., acid exposure time). In addition, hypervigilance is profoundly involved in visceral hypersensitivity [16]. The concept of hypervigilance refers to the enhanced state of attention or alertness to sensations caused by a threat [17]. As a result, symptoms can be amplified (i.e., hyperalgesia) or perceived even in the absence of noxious stimulation (i.e., allodynia). Both hypervigilance and symptom anxiety are inherently protective actions by the brain, intended to lead a person to behave in such a way that will mitigate the threat to the body (e.g., seek medical care for a chronic symptom, adhere to treatment). However, when hypervigilance and anxiety become severe, there is potential for negative impacts across patient outcomes.

Taft et al. developed and validated a novel 15-item questionnaire, the Esophageal Hypervigilance and Anxiety Scale (EHAS) that measures patients’ hypervigilance and anxiety specific to their esophageal symptoms [16]. Since then, several studies have demonstrated the impact of esophageal hypervigilance and anxiety on esophageal symptoms. In fact, EHAS score, rather than objective metrics such as acid burden, or endoscopic and histologic findings, has a greater correlation to severity of esophageal symptoms in conditions including GERD [18, 19], laryngopharyngeal reflux [20], and EoE [21]. In addition, Carlson et al. demonstrated dysphagia severity was more associated with EHAS than the lower esophageal sphincter (LES) pressure [22]. Their study showed prevalent esophageal hypervigilance and anxiety across all types of esophageal motility. Nevertheless, the impact of EHAS on esophageal symptoms across different subtypes in disorders of esophagogastric junction (EGJ) outflow remains incompletely explored [23].

The EHAS has become indispensable for clinical practice as well as research, since the recent expert consensus recommends assessing symptom-specific hypervigilance and anxiety in all patients with heartburn to determine the suitability of brain–gut behavioral therapies (BGBT) such as cognitive behavioral therapy in patients with esophageal DGBI [24]. However, the EHAS was originally developed in English and its validated Japanese version has not been available to date. Therefore, the aim of this study was to (1) develop and validate the Japanese version of EHAS and (2) examine esophageal hypervigilance and anxiety and identify factors associated with symptom severity in untreated disorders of esophagogastric junction (EGJ) outflow.

## Methods

### Study population

We recruited Japanese-speaking patients aged between 20 and 85 years who underwent high-resolution manometry (HRM) for upper gastrointestinal symptoms at six tertiary centers between November 2021 and October 2023. Verbal consent was obtained from patients after they were informed that their clinical data including patients-reported outcome questionnaires would be used in this study. Patients were excluded if they had a history of foregut surgery except endoscopic therapy or Heller myotomy, or they had severe heart, renal and liver failure. This multi-center study was performed according to the Declaration of Helsinki and was initially approved by the Osaka Metropolitan University ethics committee (#2021–192) and subsequently confirmed by other five centers’ ethical committees (Nippon Medical School, Gunma University, Tohoku University, Kyushu University, and Aichi Medical University).

### Patient-reported outcome questionnaires

EHAS

The EHAS is a 15-item questionnaire to assess anxiety and hypervigilance specifically for esophageal symptoms [16]. Each item assesses patients’ thoughts, beliefs, and attitudes toward esophageal symptoms using a 5-point Likert scale from strongly disagree (0 point) to strongly agree (4 points). The total score ranges from 0 (no anxiety and hypervigilance to esophageal symptoms) to 60 (severe anxiety and hypervigilance). The original EHAS consists of two subscales, anxiety (questions 1–9) and hypervigilance (questions 10–15).

Eckardt score

The Eckardt score is a four-item questionnaire that measures weight loss, dysphagia, chest pain, and regurgitation on a 4-point Likert scale from 0 to 3 [25]. For dysphagia, chest pain, and regurgitation, the score is rated on the basis of their frequency as follows: 0 = none, 1 = occasional, 2 = daily, 3 = each meal. Weight loss in kilograms is scored as follows: 0 = none, 1 =  < 5 kg, 2 = 5–10 kg, 3 =  > 10 kg. The total of Eckardt score ranges from 0 (no symptom) to 12 (severe symptom). An Eckardt score greater than 3 is generally regarded as an indicator for treatment in patients with achalasia [26, 27].

## Gastroesophageal reflux disease questionnaire (GerdQ)

GerdQ is a six-item questionnaire designed for the symptom-based diagnosis and management of GERD [28, 29]. GerdQ consists of four positive and two negative predictors of GERD. Positive predictors include heartburn, regurgitation, and sleep deprivation or the use of over-the-counter drugs due to reflux symptoms, whereas negative predictors include epigastric pain and nausea. On the basis of symptom frequency over the past week, each item is rated on a 4-point Likert scale as follows: 0 = 0 days, 1 = 1 day, 2 = 2–3 days, and 3 = 4–7 days for the positive predictors and reversed order score for negative predictors. Suzuki et al. previously validated the Japanese version of GerdQ and proposed a score of 8 as a cutoff value for the diagnosis of GERD in Japan [28].

## Hospital anxiety and depression scale (HADS)

The HADS is a 14-item questionnaire for the screening of generalized anxiety and depression in patients with medical conditions [30]. There are seven items for each anxiety (HADS-A) and depression (HADS-D) rated on a 4-point Liker scale from 0 to 3. In the total score for each HADS-A and HADS-D, a score of 11 or higher indicates a definite case of anxiety or depression, while a score between 8 and 10 suggests a possible case. The Japanese version of HADS has been developed and validated previously [31, 32].

### Development of the Japanese version of EHAS

According to the consensus of the International Society for Pharmacoeconomics and Outcomes Research task force [33], we developed the Japanese version of EHAS after obtaining permission from Dr. Taft owning the copyright on the original EHAS. The original EHAS was translated into Japanese by two independent gastroenterologists, and subsequently a senior gastroenterologist (YF) integrated their translations into the final Japanese version. This version was professionally backtranslated into English and confirmed by Dr. Taft that the content remained consistent with the original EHAS. We also checked the understandability of the Japanese expression in the questionnaire by cognitive debriefing with ten Japanese patients in their 20 s–80 s.

### HRM

HRM was performed to diagnose esophageal motility phenotypes according to the Chicago classification version 4.0 [34]. Esophageal motility disorders were divided into two main phenotypes, disorders of EGJ outflow and disorders of peristalsis. Disorders of EGJ outflow consist of achalasia and EGJ outflow obstruction (EGJOO). Disorders of peristalsis consist of distal esophageal spasm, hypercontractile esophagus, absent contractility, and ineffective esophageal motility. Achalasia was further categorized into three phenotypes: type I, abnormal median integrated relaxation pressure (IRP) and 100% failed peristalsis without pan-esophageal pressurization; type II, abnormal median IRP and 100% failed peristalsis with pan-esophageal pressurization in ≥ 20% swallows; and type III, abnormal median IRP, absence of intact peristalsis and ≥ 20% swallows with premature/spastic contractions [34]. Out of six centers, three centers used the same Starlet HRM system (Starmedical, Tokyo, Japan).

### Power calculation

According to the COSMIN methodology for systematic reviews of patient‐reported outcome measures (PROM), the sample size for factor analysis in the validation of PROM should be seven times the number of items and ≥ 100 [35]. In this context, 105 patients (7 × 15 items) should be adequate for EHAS. However, we aimed to recruit more than 400 patients following the previous Spanish and French EHAS validation studies [36, 37].

### Statistical analysis

We collected data pertaining to age, sex, body mass index (BMI), indication for HRM, motility phenotypes, and patient-reported outcome questionnaires. Categorical and continuous variables of demographic and clinical characteristics of study patients were expressed in number (percentage) and mean ± standard deviation (SD), respectively. If the number of missing items in an EHAS questionnaire was up to 2 (< 20% of EHAS items), each missing score was replaced with the mean value of that item score calculated from complete questionnaires. If more than three items were missing, the participants were excluded from the entire analysis.

For the total EHAS score, internal consistency was measured with Cronbach´s alpha and the Guttman split-half reliability. We evaluated the subscale structure of the Japanese EHAS using principal components factor analysis (PCFA) with Varimax rotation, extracting factors on the basis of an eigenvalue greater than 1. The Kaiser–Meyer–Olkin (KMO) value and Bartlett’s test of sphericity were calculated to check sampling adequacy for PCFA. Construct validity of the Japanese EHAS was assessed by its relationships with other questionnaires in Japanese (i.e., Eckardt score, GerdQ, and HADS) using Pearson's correlations.

To identify factors related to the severity of esophageal symptoms in patients with untreated disorders of EGJ outflow, we confined patients to those diagnosed using Starlet HRM system. This approach ensured the inclusion of consistent HRM metrics in this analysis. The univariable analysis was performed between each factor and the outcome (i.e., the Eckardt and GerdQ scores) using linear regression. In the subsequent multivariable analysis, the joint association between the factors and the outcome was evaluated by multiple regression. To limit the number of factors in the multivariable analysis, only factors with *p* < 0.2 in the univariable analyses were included.

Comparison of EHAS score between four subtypes in disorders of EGJ outflow was performed using one-way ANOVA. The relationships between EHAS and other factors were assessed using Pearson’s correlations.

*P* < 0.05 was considered statistically significant. Statistical analyses were performed using SPSS version 22 (Chicago, IL).

## Results

### Characteristics of study patients

Ultimately, 432 patients (median age 55.7 years old, female 54.9%) were analyzed after the exclusion of 5 patients due to one inadequate HRM test and four incomplete responses to questionnaires. Table 1 shows the demographic and clinical characteristics of study patients (data by center is shown in Supplementary Table 1). The most common symptom for HRM evaluation was dysphagia (47.7%), followed by reflux symptoms and chest pain. The most common motility phenotype was normal motility accounting for 35.9%. In esophageal motility disorders, achalasia was predominant (127/277, 45.8%) with IEM (51/277, 18.4%) being the next most frequently encountered phenotype.

**Table 1:** Demographic and clinical characteristics of the study patients

	*N* = 432
Age (y.o.)	55.7 ± 16.3
Female	237 (54.9%)
BMI (kg/m^2^)	21.8 ± 3.8
Indication for HRM	
Dysphagia	206 (47.7%)
Reflux symptoms	99 (22.9%)
Chest pain	36 (8.3%)
Pharyngeal discomfort	19 (4.4%)
Nausea/vomiting	15 (3.5%)
Post-POEM evaluation	14 (3.2%)
Dyspepsia	5 (1.2%)
Others	38 (8.8%)
Motility phenotype	
Normal	155 (35.9%)
Achalasia (type I/II/III)	127 (30/86/11) (29.4%)
IEM	51 (11.8%)
Post-POEM/Post Heller–Dor	39 (33/6) (9.0%)
Absent contractility	26 (6.0%)
EGJOO	17 (3.9%)
DES	9 (2.1%)
Hypercontractile esophagus	5 (1.2%)
Unclassified	3 (0.7%)
Patient-reported outcome questionnaires	
EHAS	31.2 ± 15.1
Anxiety	18.4 ± 9.6
Hypervigilance	12.8 ± 6.6
Eckardt score	3.9 ± 2.6
GerdQ	6.1 ± 3.6
HADS anxiety	6.4 ± 4.1
HADS depression	6.7 ± 3.7

*BMI* Body mass index, *HRM* High-resolution manometry, *GERD* Gastroesophageal reflux disease, *POEM* Per-oral endoscopic myotomy, *IEM* Ineffective esophageal motility, *EGJOO* Esophagogastric junction outflow obstruction, *DES* Distal esophageal spasm, *EHAS* Esophageal hypervigilance and anxiety scale; *GerdQ* Gastroesophageal reflux disease questionnaire, *HADS* Hospital anxiety and depression scale

Reflux symptoms included heartburn, regurgitation, and belching

Pharyngeal discomfort included odynophagia

Patient-reported outcome questionnaires demonstrated that 213 patients (49.3%) and 155 patients (35.9%) had abnormal Eckardt (> 3) and GerdQ (> 7) scores, respectively. According to HADS score, 76 patients (17.6%) and 72 patients (16.7%) had definite anxiety and depression, respectively.

### Factor analysis of the Japanese EHAS

This study collected adequate sample for PCFA analysis as the KMO value was 0.939 and Bartlett’s test of sphericity was statistically significant (p < 0.001). PCFA yielded two subscales in the Japanese EHAS similar to the original version: factor 1 for anxiety (question 1–9, 13, 15) and factor 2 (question 9 to 15) for hypervigilance (Table 2). Factors 1 and 2 accounted for 55.4% and 9.9% of the variance in the Japanese EHAS score, respectively. Item 15, which measures how much the person monitors their symptoms, loaded slightly more strongly on the anxiety subscale and item 9, which measures how anxiously the person wants their symptoms to go away, loaded slightly more strongly on the hypervigilance subscale. As such, the Japanese EHAS subscales can be scored in the same manner as the original English version.

**Table 2:** Factor loadings of the Japanese version of EHAS after principal component factor analysis with varimax rotation

EHAS items	Original EHAS component	Factor 1	Factor 2
		eigenvalue = 8.30	eigenvalue = 1.49
Question 1	Anxiety	0.757	
Question 2	Anxiety	0.794	
Question 3	Anxiety	0.807	
Question 4	Anxiety	0.785	
Question 5	Anxiety	0.773	
Question 6	Anxiety	0.738	
Question 7	Anxiety	0.661	
Question 8	Anxiety	0.676	
Question 9	Anxiety	0.472	0.508
Question 10	Hypervigilance		0.846
Question 11	Hypervigilance		0.870
Question 12	Hypervigilance		0.727
Question 13	Hypervigilance	0.460	0.667
Question 14	Hypervigilance		0.707
Question 15	Hypervigilance	0.646	0.522

*EHAS* Esophageal hypervigilance and anxiety scale

Only factor loadings > 0.4 are displayed

### Reliability and validity of the Japanese EHAS

The Japanese EHAS demonstrated excellent internal consistency (Cronbach’s α = 0.942) and split-half reliability (Guttman Static = 0.88). In addition, there was significant inter-item correlation in the Japanese EHAS (*p* < 0.001), in which correlation coefficient ranged from 0.241 (Q7 and Q11) to 0.795 (Q2 and Q3). This suggests that the Japanese EHAS demonstrated internal consistency with each item independently contributing to the overall construct measurement of the questionnaire. The Japanese EHAS significantly correlated with other patient-reported questionnaires pertaining to esophageal symptoms such as the Eckardt score, GerdQ, and HADS anxiety and depression (*p* < 0.001) (Table 3), suggesting good construct validity. Similarly, significant correlations with other patient-reported questionnaires were observed in each anxiety and hypervigilance subscale of EHAS (*p* < 0.01).

**Table 3:** Correlation between patient-reported outcome questionnaires

	EHAS total	EHAS anxiety	EHAS hypervigilance	Eckardt score	GerdQ	HADS anxiety	HADS depression
EHAS total	–						
EHAS anxiety	0.954^*^	–					
EHAS hypervigilance	0.900^*^	0.727^*^	–				
Eckardt score	0.545^*^	0.512^*^	0.502^*^	–			
GerdQ	0.303^*^	0.319^*^	0.228^*^	0.333^*^	–		
HADS anxiety	0.457^*^	0.447^*^	0.393^*^	0.232^*^	0.160^*^	–	
HADS depression	0.213^*^	0.237^*^	0.142^*^	0.091	0.165^*^	0.540^*^	-

*EHAS* Esophageal hypervigilance and anxiety scale, *GerdQ* Gastroesophageal reflux questionnaire, *HADS* Hospital anxiety and depression scale

^*^p < 0.01

### Relationship between the Japanese EHAS and the severity of esophageal symptoms

**Fig. 1:**
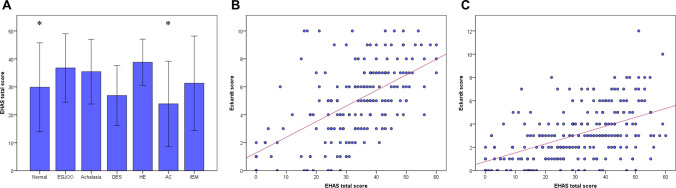
EHAS score between esophageal motility subtypes. Esophageal hypervigilance and anxiety were widely observed in every esophageal motility subtype, in which patients with normal motility or absent contractility had significantly a lower EHAS score compared to those with achalasia **A**. The EHAS scores significantly correlated with the Eckardt score in both patients with **B** and without disorders of EGJ outflow **C**.* *p* < 0.05 vs achalasia. *EHAS* Esophageal Hypervigilance and Anxiety Scale, *EGJOO* Esophagogastric junction outflow obstruction, *DES* Distal esophageal spasm, *HE* Hypercontractile esophagus, *AC* Absent contractility, *IEM* Ineffective esophageal motility

Esophageal hypervigilance and anxiety were observed in every type of esophageal motility, in which patients with normal motility or absent contractility had significantly lower EHAS score than those with achalasia (*p* < 0.05) (Fig. 1A). EHAS score moderately correlated to Eckardt score (i.e., the severity of esophageal symptoms) in those with (*N* = 183) (*r* = 0.575; 95% CI, 0.469–0.665, *p* < 0.001) and without disorders of EGJ outflow (*N* = 249) (r = 0.536; 95% CI, 0.441–0.619, *p* < 0.001) (Fig. 1B, C).

### Factors associated with esophageal symptom severity in untreated disorders of EGJ outflow

**Fig. 2:**
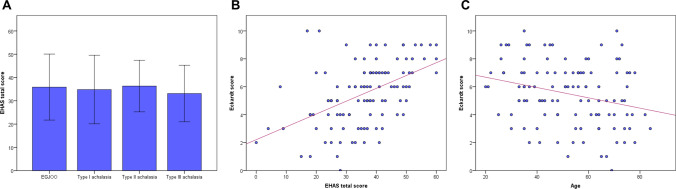
Correlation between the Eckardt score, the EHAS, and age in untreated disorders of EGJ outflow. The EHAS score does not differ between the four phenotypes in untreated disorders of EGJ outflow **A**. The Eckardt score demonstrated positive and negative correlation with **B** the EHAS score and **C** age, respectively. *EHAS* Esophageal Hypervigilance and Anxiety Scale, *EGJ* esophagogastric junction, *EGJOO* Esophagogastric junction outflow obstruction

Table 4 shows the clinical characteristics of 113 patients with untreated disorders of EGJ outflow evaluated using Starlet HRM system that consisted of 9 EGJOO and 104 achalasia. Type II achalasia (5.9 ± 1.9) had a relatively high Eckardt score compared to EGJOO (4.6 ± 2.0), type I (4.8 ± 3.0) and type III achalasia (4.8 ± 1.9), although it did not reach statistically significant difference (*p* = 0.075). While the total scores of EHAS were comparable between the four groups (*p* = 0.880) (Fig. 2A). Univariable analysis identified age, BMI, IRP, basal LES pressure, and the EHAS score as factors associated with the Eckardt score. In multivariable analysis, age, type II achalasia, and the EHAS score were independently and significantly associated with the Eckardt score (i.e., more severe esophageal symptom), while symptom duration, HRM metrics, generalized anxiety, and depression were non-significant. The Eckardt score was positively and moderately correlated to the EHAS score (Fig. 2B). In contrast, the Eckardt score demonstrated a negative and weak association with age (Fig. 2C).

**Table 4:** Factors associated with the Eckardt score in untreated disorders of EGJ outflow

Variables		Univariable analysis		Multivariable analysis	
	*N* = 113	Coefficient (95% CI)	P value	Coefficient (95% CI)	*P* value
Age	52.0 ± 16.4	−0.04 (−0.06 to −0.01)	0.004	−0.03 (−0.05 to −0.01)	0.011
Male	50 (44.2%)	0.19 (−0.65 to 1.03)	0.660		
BMI	21.1 ± 3.7	-−0.13 (−0.24 to −0.01)	0.027	−0.09 (−0.19 to 0.01)	0.088
Any psychiatric disorders ^a^	1 (0.9%)	3.54 (−0.88 to 7.95)	0.115	0.97 (−2.81 to 4.74)	0.612
Symptom duration (month)^b^	331 ± 2297	−0.02 (−0.03 to −0.002)	0.076	−0.01 (−0.03 to 0.002)	0.095
Motility phenotype					
EGJOO	9 (8.0%)	Reference			
Type I achalasia	22 (19.5%)	0.26 (−1.46 to 1.98)	0.763		
Type II achalasia	74 (65.5%)	1.33 (−0.19 to 2.87)	0.087	0.83 (0.08 to 1.59)	0.032
Type III achalasia	8 (7.1%)	0.19 (−1.91 to 2.31)	0.856		
HRM metrics					
IRP (mmHg)	33.0 ± 12.8	0.04 (0.01 to 0.07)	0.009	0.02 (−0.02 to 0.06)	0.393
Basal LES pressure (mmHg)	34.7 ± 14.8	0.04 (0.01 to 0.06)	0.01	0.01 (−0.03 to 0.04)	0.658
PRO questionnaires					
EHAS total	35.8 ± 12.0	0.09 (0.06 to 0.12)	< 0.001	0.09 (0.05 to 0.12)	< 0.001
HADS anxiety	6.8 ± 4.0	0.08 (−0.03 to 0.18)	0.138	−0.08 (−0.18 to 0.02)	0.101
HADS depression	6.0 ± 3.4	0.05 (−0.07 to 0.18)	0.402		

*BMI* Body mass index, *HRM* High-resolution manometry, *IRP* Integrated relaxation pressure, *LES* Lower esophageal sphincter, *DCI* Distal contractile integral, *PRO* Patient-reported outcome, *EHAS* Esophageal hypervigilance and anxiety scale, *HADS* Hospital anxiety and depression scale

^a^ Psychiatric disorders include anxiety, depression, bipolar disorder, schizophrenia, or panic disorder

^b^ Regression coefficients given for a 100-unit increase in variable

For GerdQ score, univariable analysis found a significant association with the EHAS score, and this relationship persisted in the multivariable analysis (Supplementary Table 2).

## Discussion

Visceral hypersensitivity is deeply involved in the severity of GI symptoms by amplifying signals transmitted from the peripheral nerve endings to the central nervous system [38]. This hypersensitivity can amplify esophageal sensations, causing some patients to experience distressing symptoms both in the presence of and absence of physiological or peripheral stimulation. Hypervigilance and symptom-specific anxiety are important psychological factors that may contribute to visceral hypersensitivity by putting patients on alert for visceral sensation [16]. Functional heartburn is one of the visceral hypersensitivity-driven esophageal conditions where patients experience reflux symptoms despite a negative reflux-symptom association [38].

If patients with functional heartburn are highly hypervigilant and anxious pertaining to their symptoms, psychological intervention should be a reasonable option for visceral desensitization. A recent expert consensus recommends psychosocial assessment inclusive of symptom-specific hypervigilance and anxiety and health-related quality of life for all patients with heartburn to determine the application of BGBT intervention [24]. There are several generalizable (i.e., not disease specific) tools to assess general hypervigilance and anxiety (i.e., the HADS and the Somatosensory Amplification Scale) [39]. However, prior to the advent of EHAS, no questionnaires were available for measuring those specific to esophageal symptoms [16].

The EHAS has been increasingly recognized as an essential tool in the management of esophageal disorders [24], and thus we developed its Japanese version. This study confirmed the reliability and construct validity of the Japanese EHAS using standardized psychometric assessment approaches, including forward and backward translation by native Japanese speakers, and concurrent administration with other validated Japanese questionnaires to assess construct validity. Our findings suggest that the total EHAS score can be used in the future international studies between Japan and English-speaking countries. The Japanese EHAS was composed of two subscales similar to the original EHAS. However, PCFA classified two of the hypervigilance-related items in the original EHAS (i.e., Questions 13 “I focus on esophageal sensations” and 15 “I keep track of my symptom levels”) into anxiety subscale in the Japanese EHAS. Similarly, one originally anxiety-related item was included in the hypervigilance subscale in the Japanese EHAS (Question 9 “I anxiously want the symptoms to go away”). This discordance might be due to cultural differences in the perception of symptoms between Japan and the USA. In addition, the different proportions of motility phenotypes could have affected the results. This study included a larger proportion of patients with disorders of EGJ outflow (42.3%) compared to the validation study of the original EHAS (30.4%) [16]. However, since the differences in factor loadings were small, we can recommend using both the full scale and subscale scores of the Japanese EHAS with some caution. Future studies utilizing the Japanese EHAS should assess the subscales for proper factor loading and in the event of discrepancies, default to using the total EHAS score only.

We found that esophageal hypervigilance and symptom-specific anxiety are prevalent in every type of esophageal motility in line with previous studies [36, 37]. In other words, the EHAS score depends not on objective severity of an esophageal motility disorder. Instead, the EHAS was significantly associated with symptom severity (i.e., Eckardt and GerdQ scores). These positive correlations indicate that the Japanese EHAS can be applied to a wide range of esophageal symptoms, such as dysphagia, chest pain, and reflux symptoms, to assess the need for psychological intervention. It is likely that the two factors, esophageal hypervigilance and symptom-specific anxiety, and symptom severity bidirectionally interact with each other and end up creating a vicious cycle. Further, Paterson, et al. demonstrated that repeated esophageal distension amplifies chest pain as the number of distension increases [40]. This finding suggests that repetitive noxious stimuli develop hypervigilance and anxiety regarding esophageal symptoms, which in turn amplify their intensity (i.e., hyperalgesia). Such acquired responses to esophageal symptom are unlikely to subside swiftly [41]. If a single episode of catastrophic esophageal symptoms causes patients to develop hyperalgesia and/or allodynia, those conditions may persist even in the absence of further episodes [41]. In this instance, psychological intervention should be considered for symptom relief [42, 43].

The main mechanism of esophageal symptoms probably differs between disorders of EGJ outflow and disorders of peristalsis given discrete physiological characteristics (e.g., esophageal bolus transit), and for this reason esophageal hypervigilance and symptom-specific anxiety could differently influence the symptom severity between the two entities. Factors associated with symptom severity, inclusive of EHAS, have not been fully investigated in a large cohort of subtyped achalasia patients. Therefore, we investigated the correlation between EHAS and symptom severity, focusing on patients with untreated disorders of EGJ outflow. We found that higher EHAS score, as well as younger age, correlated with higher Eckardt score, whereas objective physiological metrics such as IRP and basal LES pressure did not. Negative association between symptom severity and objective HRM metrics in achalasia is consistent with a previous achalasia study by Nicodème, et al. [23]. In addition, young achalasia patients tend to have chest pain [44].

This study has several strengths. The Japanese EHAS was developed according to consensus guidelines [33], and the validation was performed using a large number of patients who underwent HRM at six tertiary centers in Japan. However, there are also a few limitations. In the test of construct validity of the Japanese EHAS, only the Eckardt score has not been validated in Japanese, although the Eckardt score has been commonly used in clinical practice as well as research in Japan. We did not use questionnaires to assess health-related quality of life for construct validity, unlike the original EHAS study. In the analysis of esophageal symptoms severity-related factors, the number of patients with EGJOO or type III achalasia was not adequate. In addition, we did not include other potential contributors to dysphagia and chest pain including esophageal morphology, bolus clearance [45], and superficial mucosal afferent nerves [9]. Lack of data on lifestyles could also influence the outcome of the relationship between the EHAS score and esophageal symptoms.

In conclusion, the Japanese EHAS is a reliable and valid questionnaire to assess hypervigilance and anxiety specific to esophageal symptoms. The EHAS score was profoundly associated with the severity of esophageal symptoms, and thus a useful tool to evaluate psychological status in patients with esophageal disorders for both clinical and research purposes. Psychosocial assessment is essential, especially for the management of esophageal DGBI [24]. However, there has been little data pertaining to how to use EHAS to judge the appropriateness of brain–gut behavioral therapies. Future study is warranted to demonstrate a cutoff value of EHAS for selection of patients who are likely to respond to these treatments.

## Supplementary Information

Below is the link to the electronic supplementary material.

Supplementary file1 (DOCX 25 KB)

## References

[1] Fujiwara Y, Arakawa T. Epidemiology and clinical characteristics of GERD in the Japanese population. J Gastroenterol. 2009;44:518–34. 10.1007/s00535-009-0047-5. 19365600 10.1007/s00535-009-0047-5

[2] Iwakiri K, Fujiwara Y, Manabe N, et al. Evidence-based clinical practice guidelines for gastroesophageal reflux disease 2021. J Gastroenterol. 2022;57:267–85. 10.1007/s00535-022-01861-z. 35226174 10.1007/s00535-022-01861-z PMC8938399

[3] Fujiwara Y. Recent epidemiology of GERD in the Japanese population. Nihon Shokakibyo Gakkai Zasshi. 2017;114:1781–9. 10.11405/nisshoshi.114.1781. 28978878 10.11405/nisshoshi.114.1781

[4] Peery AF, Crockett SD, Murphy CC, et al. Burden and cost of gastrointestinal, liver, and pancreatic diseases in the united states: update 2021. Gastroenterology. 2022;162:621–44. 10.1053/j.gastro.2021.10.017. 34678215 10.1053/j.gastro.2021.10.017 PMC10756322

[5] Sawada A, Sifrim D, Fujiwara Y. Esophageal reflux hypersensitivity: a comprehensive review. Gut Liver. 2023;17:831–42. 10.5009/gnl220373. 36588526 10.5009/gnl220373 PMC10651372

[6] Woodland P, Al-Zinaty M, Yazaki E, et al. In vivo evaluation of acid-induced changes in oesophageal mucosa integrity and sensitivity in non-erosive reflux disease. Gut. 2013;62:1256–61. 10.1136/gutjnl-2012-302645. 22722617 10.1136/gutjnl-2012-302645

[7] Norita K, Asanuma K, Koike T, et al. Impaired mucosal integrity in proximal esophagus is involved in development of proton pump inhibitor-refractory nonerosive reflux disease. Digestion. 2021;102:404–14. 10.1159/000508661. 32784296 10.1159/000508661

[8] Woodland P, Shen Ooi JL, Grassi F, et al. Superficial esophageal mucosal afferent nerves may contribute to reflux hypersensitivity in nonerosive reflux disease. Gastroenterology. 2017;153:1230–9. 10.1053/j.gastro.2017.07.017. 28734832 10.1053/j.gastro.2017.07.017

[9] Sawada A, Zhang M, Ustaoglu A, et al. Superficial oesophageal mucosal innervation may contribute to severity of symptoms in oesophageal motility disorders. Aliment Pharmacol Ther. 2024;59:100–12. 10.1111/apt.17773. 37845817 10.1111/apt.17773

[10] Schey R, Dickman R, Parthasarathy S, et al. Sleep deprivation is hyperalgesic in patients with gastroesophageal reflux disease. Gastroenterology. 2007;133:1787–95. 10.1053/j.gastro.2007.09.039. 18054551 10.1053/j.gastro.2007.09.039

[11] Fujiwara Y, Arakawa T, Fass R. Gastroesophageal reflux disease and sleep disturbances. J Gastroenterol. 2012;47:760–9. 10.1007/s00535-012-0601-4. 22592763 10.1007/s00535-012-0601-4

[12] Hoshikawa Y, Momma E, Kawami N, et al. Lemborexant attenuates regurgitation without worsening objective parameters on reflux monitoring in patients with gastroesophageal reflux disease and insomnia: a single-arm proof-of-concept study. Digestion. 2023;104:438–45. 10.1159/000531412. 37429270 10.1159/000531412

[13] Okuyama M, Nakahara K, Iwakura N, et al. Factors associated with potassium-competitive acid blocker non-response in patients with proton pump inhibitor-refractory gastroesophageal reflux disease. Digestion. 2017;95:281–7. 10.1159/000475658. 28501868 10.1159/000475658

[14] Kessing BF, Bredenoord AJ, Saleh CM, et al. Effects of anxiety and depression in patients with gastroesophageal reflux disease. Clin Gastroenterol Hepatol : Off Clin Pract J Am Gastroenterol Assoc. 2015;13:1089-95.e1. 10.1016/j.cgh.2014.11.034. 10.1016/j.cgh.2014.11.034 25496817

[15] Okuyama M, Takaishi O, Nakahara K, et al. Associations among gastroesophageal reflux disease, psychological stress, and sleep disturbances in Japanese adults. Scand J Gastroenterol. 2017;52:44–9. 10.1080/00365521.2016.1224383. 27571846 10.1080/00365521.2016.1224383

[16] Taft TH, Triggs JR, Carlson DA, et al. Validation of the oesophageal hypervigilance and anxiety scale for chronic oesophageal disease. Aliment Pharmacol Ther. 2018;47:1270–7. 10.1111/apt.14605. 29528128 10.1111/apt.14605 PMC5897170

[17] Hollins M, Walters S. Experimental hypervigilance changes the intensity/unpleasantness ratio of pressure sensations: evidence for the generalized hypervigilance hypothesis. Exp Brain Res. 2016;234:1377–84. 10.1007/s00221-015-4541-0. 26724932 10.1007/s00221-015-4541-0

[18] Wong MW, Liu TT, Yi CH, et al. Oesophageal hypervigilance and visceral anxiety relate to reflux symptom severity and psychological distress but not to acid reflux parameters. Aliment Pharmacol Ther. 2021;54:923–30. 10.1111/apt.16561. 34383968 10.1111/apt.16561

[19] Guadagnoli L, Yadlapati R, Taft T, et al. Esophageal hypervigilance is prevalent across gastroesophageal reflux disease presentations. Neurogastroenterol Motil. 2021;33: e14081. 10.1111/nmo.14081. 33432708 10.1111/nmo.14081 PMC8272741

[20] Wong MW, Hsiao SH, Wang JH, et al. Esophageal hypervigilance and visceral anxiety contribute to symptom severity of laryngopharyngeal reflux. Am J Gastroenterol. 2023;118:786–93. 10.14309/ajg.0000000000002151. 36693025 10.14309/ajg.0000000000002151

[21] Taft TH, Carlson DA, Simons M, et al. Esophageal hypervigilance and symptom-specific anxiety in patients with eosinophilic esophagitis. Gastroenterology. 2021;161:1133–44. 10.1053/j.gastro.2021.06.023. 34153298 10.1053/j.gastro.2021.06.023 PMC8463417

[22] Carlson DA, Gyawali CP, Roman S, et al. Esophageal hypervigilance and visceral anxiety are contributors to symptom severity among patients evaluated with high-resolution esophageal manometry. Am J Gastroenterol. 2020;115:367–75. 10.14309/ajg.0000000000000536. 31990697 10.14309/ajg.0000000000000536 PMC7071929

[23] Nicodème F, de Ruigh A, Xiao Y, et al. A comparison of symptom severity and bolus retention with Chicago classification esophageal pressure topography metrics in patients with achalasia. Clin Gastroenterol Hepatol : Off Clin Pract J Am Gastroenterol Assoc. 2013. 10.1016/j.cgh.2012.10.015. 10.1016/j.cgh.2012.10.015 PMC3552153 23078890

[24] Guadagnoli L, Yadlapati R, Pandolfino J, et al. Behavioral therapy for functional heartburn: recommendation statements. Clin Gastroenterol Hepatol : Off Clin Pract J Am Gastroenterol Assoc. 2024;22(1709–18): e3. 10.1016/j.cgh.2024.03.004. 10.1016/j.cgh.2024.03.004 PMC11272445 38518891

[25] Eckardt VF, Aignherr C, Bernhard G. Predictors of outcome in patients with achalasia treated by pneumatic dilation. Gastroenterology. 1992;103:1732–8. 10.1016/0016-5085(92)91428-7. 1451966 10.1016/0016-5085(92)91428-7

[26] Ponds FA, Fockens P, Lei A, et al. Effect of peroral endoscopic myotomy vs pneumatic dilation on symptom severity and treatment outcomes among treatment-naive patients with achalasia: a randomized clinical trial. JAMA. 2019;322:134–44. 10.1001/jama.2019.8859. 31287522 10.1001/jama.2019.8859 PMC6618792

[27] Saleh CMG, Familiari P, Bastiaansen BAJ, et al. The efficacy of peroral endoscopic myotomy vs pneumatic dilation as treatment for patients with achalasia suffering from persistent or recurrent symptoms after laparoscopic heller myotomy: a randomized clinical trial. Gastroenterology. 2023;164(1108–18): e3. 10.1053/j.gastro.2023.02.048. 10.1053/j.gastro.2023.02.048 36907524

[28] Suzuki H, Matsuzaki J, Okada S, et al. Validation of the GerdQ questionnaire for the management of gastro-oesophageal reflux disease in Japan. United European Gastroenterol J. 2013;1:175–83. 10.1177/2050640613485238. 24917957 10.1177/2050640613485238 PMC4040759

[29] Jones R, Junghard O, Dent J, et al. Development of the GerdQ, a tool for the diagnosis and management of gastro-oesophageal reflux disease in primary care. Aliment Pharmacol Ther. 2009;30:1030–8. 10.1111/j.1365-2036.2009.04142.x. 19737151 10.1111/j.1365-2036.2009.04142.x

[30] Zigmond AS, Snaith RP. The hospital anxiety and depression scale. Acta Psychiatr Scand. 1983;67:361–70. 10.1111/j.1600-0447.1983.tb09716.x. 6880820 10.1111/j.1600-0447.1983.tb09716.x

[31] Higashi A, Yashiro H, Kiyota K, et al. Validation of the hospital anxiety and depression scale in a gastro-intestinal clinic. Nihon Shokakibyo Gakkai Zasshi. 1996;93:884–92. 8986079

[32] Hatta H, Higashi A, Yashiro H, et al. A validation of the hospital anxiety and depression scale. Japanese J Psychosomatic Med. 1998;38:309–15.

[33] Wild D, Grove A, Martin M, et al. Principles of good practice for the translation and cultural adaptation process for patient-reported outcomes (pro) measures: report of the ispor task force for translation and cultural adaptation. Value Health. 2005;8:94–104. 10.1111/j.1524-4733.2005.04054.x. 15804318 10.1111/j.1524-4733.2005.04054.x

[34] Yadlapati R, Kahrilas PJ, Fox MR, et al. Esophageal motility disorders on high-resolution manometry: Chicago classification version 40((c)). Neurogastroenterol Motil. 2021. 10.1111/nmo.14058. 33373111 10.1111/nmo.14058 PMC8034247

[35] Prinsen CAC, Mokkink LB, Bouter LM, et al. COSMIN guideline for systematic reviews of patient-reported outcome measures. Qual Life Res. 2018;27:1147–57. 10.1007/s11136-018-1798-3. 29435801 10.1007/s11136-018-1798-3 PMC5891568

[36] Cisternas D, Taft T, Carlson DA, et al. The Spanish version of the esophageal hypervigilance and anxiety score shows strong psychometric properties: Results of a large prospective multicenter study in Spain and Latin America. Neurogastroenterol Motil. 2021;33:e14102. 10.1111/nmo.14102. 33580617 10.1111/nmo.14102

[37] Roman S, Guadagnoli LA, Hastier A, et al. Validation of the French version of the esophageal hypervigilance and anxiety scale. Clin Res Hepatol Gastroenterol. 2021;45: 101672. 10.1016/j.clinre.2021.101672. 33722776 10.1016/j.clinre.2021.101672

[38] Aziz Q, Fass R, Gyawali CP, et al. Functional esophageal disorders. Gastroenterology. 2016. 10.1053/j.gastro.2016.02.012. 27144625 10.1053/j.gastro.2016.02.012

[39] Barsky AJ, Wyshak G, Klerman GL. The somatosensory amplification scale and its relationship to hypochondriasis. J Psychiatr Res. 1990;24:323–34. 2090830 10.1016/0022-3956(90)90004-a

[40] Paterson WG, Wang H, Vanner SJ. Increasing pain sensation to repeated esophageal balloon distension in patients with chest pain of undetermined etiology. Dig Dis Sci. 1995;40:1325–31. 10.1007/BF02065546. 7781455 10.1007/BF02065546

[41] Ceunen E, Zaman J, Weltens N, et al. Learned fear of gastrointestinal sensations in healthy adults. Clin gastroenterol Hepatol : Off Clin Pract J Am Gastroenterol Assoc. 2016;14(1552–8): e2. 10.1016/j.cgh.2016.04.035. 10.1016/j.cgh.2016.04.035 27155550

[42] Gasiorowska A, Navarro-Rodriguez T, Dickman R, et al. Clinical trial: the effect of Johrei on symptoms of patients with functional chest pain. Aliment Pharmacol Ther. 2009;29:126–34. 10.1111/j.1365-2036.2008.03859.x. 18945261 10.1111/j.1365-2036.2008.03859.x

[43] Gonzalez-Ibarra F, Cruz-Ruiz M, Murillo Llanes J, et al. The role of psychological factors in noncardiac chest pain of esophageal origin. J Neurogastroenterol Motil. 2024;30:272–80. 10.5056/jnm23166. 38972864 10.5056/jnm23166 PMC11238108

[44] Eckardt VF, Stauf B, Bernhard G. Chest pain in achalasia: patient characteristics and clinical course. Gastroenterology. 1999;116:1300–4. 10.1016/s0016-5085(99)70493-2. 10348812 10.1016/s0016-5085(99)70493-2

[45] Carlson DA, Beveridge CA, Lin Z, et al. Improved assessment of bolus clearance in patients with achalasia using high-resolution impedance manometry. Clin Gastroenterol Hepatology : Off Clin Pract J Am Gastroenterol Assoc. 2018;16(672–80): e1. 10.1016/j.cgh.2017.11.019. 10.1016/j.cgh.2017.11.019 PMC5911237 29155168

