# Supplementary material for: Validation of the Japanese version of the Esophageal Hypervigilance and Anxiety Scale for esophageal symptoms
Source: J Gastroenterol. 2024 Dec 9;60(3):265–74. doi: 10.1007/s00535-024-02193-w (PMC11879752; doi:10.1007/s00535-024-02193-w)
Supplement: Supplementary file 1 — Supplementary file1 (DOCX 25 KB) [file 535_2024_2193_MOESM1_ESM.docx]

Supplementary Table 1. Demographic and clinical characteristics of study patients by center

|  | Osaka Metropolitan University | Nippon Medical School | Gunma University | Tohoku University | Kyushu University | Aichi Medical University | P value |
| --- | --- | --- | --- | --- | --- | --- | --- |
| Number of patients | 202 | 89 | 59 | 32 | 30 | 20 |  |
| Age (y.o.) | 55.6 ± 17.1 | 54.2 ± 17.0 | 57.2 ± 13.7 | 52.1 ± 14.6 | 61.0 ± 12.9 | 57.5 ± 17.7 | 0.287 |
| Female | 108 (53.5%) | 62 (69.7%) | 27 (45.8%) | 15 (46.9%) | 16 (53.3%) | 9 (45.0%) | 0.040 |
| BMI (kg/m^2^) | 21.9 ± 3.8 | 21.1 ± 3.9 | 21.3 ± 3.4 | 23.4 ± 4.1 | 21.4 ± 3.5 | 21.6 ± 3.4 | 0.075 |
| Indication for HRM |  |  |  |  |  |  | <0.001 |
| Dysphagia | 113 (56.0%) | 31 (34.8%) | 19 (32.2%) | 11 (34.3%) | 20 (66.7%) | 12 (60.0%) |  |
| Reflux symptoms | 36 (17.8%) | 31 (34.8%) | 6 (10.2%) | 15 (46.9%) | 6 (20.0%) | 5 (25.0%) |  |
| Chest pain | 20 (9.9%) | 7 (7.9%) | 5 (8.5%) | 3 (9.4%) | 0 (0.0%) | 1 (5.0%) |  |
| Others | 33 (16.3%) | 20 (22.5%) | 29 (49.1%) | 3 (9.4%) | 4 (13.3%) | 2 (10.0%) |  |

Supplementary Table 2. Factors associated with the GerdQ score in untreated disorders of EGJ outflow

| Variables | Univariable analysis | | Multivariable analysis | |
| --- | --- | --- | --- | --- |
|  | Coefficient (95% CI) | P value | Coefficient (95% CI) | P value |
| Age | 0.01 (-0.03 to 0.05) | 0.793 |  |  |
| Male | -0.47 (-1.77 to 0.82) | 0.472 |  |  |
| BMI | -0.14 (-0.32 to 0.03) | 0.096 | -0.08 (-0.26 to 0.10) | 0.400 |
| Any psychiatric disorders ^a^ | 3.13 (-3.73 to 9.98) | 0.368 |  |  |
| Symptom duration (month) ^b^ | -0.01 (-0.04 to 0.01) | 0.302 |  |  |
| Motility phenotype |  |  |  |  |
| EGJOO | Reference |  |  |  |
| Type I achalasia | -0.26 (-2.97 to 2.44) | 0.848 |  |  |
| Type II achalasia | -0.50 (-2.91 to 1.91) | 0.683 |  |  |
| Type III achalasia | -2.32 (-5.64 to 1.00) | 0.169 | -1.51 (-3.97 to 0.95) | 0.226 |
| HRM metrics |  |  |  |  |
| IRP (mmHg) | 0.03 (-0.02 to 0.08) | 0.194 | 0.01 (-0.07 to 0.08) | 0.890 |
| Basal LES pressure (mmHg) | 0.04 (-0.004 to 0.08) | 0.076 | 0.03 (-0.03 to 0.09) | 0.373 |
| PRO questionnaires |  |  |  |  |
| EHAS total | 0.07 (0.01 to 0.12) | 0.015 | 0.07 (0.01 to 0.12) | 0.021 |
| HADS anxiety | -0.03 (-0.20 to 0.13) | 0.694 |  |  |
| HADS depression | -0.13 (-0.32 to 0.06) | 0.177 | -0.17 (-0.37 to 0.02) | 0.085 |

Abbreviations: GerdQ, gastroesophageal reflux disease questionnaire; BMI, body mass index; HRM, high-resolution manometry; IRP, integrated relaxation pressure; LES, lower esophageal sphincter; DCI, distal contractile integral; PRO, patient-reported outcome; EHAS, esophageal hypervigilance and anxiety scale; HADS, Hospital Anxiety and Depression Scale.

^a^ Psychiatric disorders include anxiety, depression, bipolar disorder, schizophrenia, or panic disorder

^b^ Regression coefficients given for a 100-unit increase in variable
